# Taxoid profile in endophytic fungi isolated from *Corylus avellana*, introduces potential source for the production of Taxol in semi-synthetic approaches

**DOI:** 10.1038/s41598-022-13602-6

**Published:** 2022-06-07

**Authors:** Narjes Mohammadi Ballakuti, Faezeh Ghanati, Hassan Zare-Maivan, Mozhgan Alipour, Mahtab Moghaddam, Parviz Abdolmaleki

**Affiliations:** 1 Department of Plant Biology, Faculty of Biological Science, Tarbiat Modares University, POB: 14115-154, Tehran, Iran; 2 Department of Biophysics, Faculty of Biological Science, Tarbiat Modares University, POB: 14115-154, Tehran, Iran

**Keywords:** Biological techniques, Drug discovery, Plant sciences

## Abstract

Taxol (Paclitaxel) and its derivative taxanes are widely used in chemotherapy and treatment of different types of cancer. Although the extracted taxanes from *Taxus sp.* are currently used in semi-synthetic production of Taxol, providing alternative always available sources is still a main concern. Due to availability and fast growth rate, microorganisms are much potent alternative sources for taxanes. In the present study, 249 endophytic fungi were isolated from *Corylus avellana* at six different locations of Iran, among which 18 species were capable to produce taxanes. Genotyping analysis indicated that 17 genera were ascomycetes but only one basidiomycete. Seven taxanes were detected and quantified in solid and suspension cultures by HPLC and their structures were confirmed by LC-Mass analysis. Among endophytes, CA7 had all 7 taxoids and CA1 had the highest Taxol yield. In 78% of endophytes transferring to liquid media was accompanied by increase of taxanes yield and increased taxan production and its release to media up to 90%. Evaluation of cytotoxicity indicated that extracts of all isolated fungi were lethal to MCF7 cells. Since endophytes produced remarkable amounts of taxanes, they can be suggested as alternative inexpensive and easily available resources for Taxol production in semi-synthesis plans.

## Introduction

Taxanes are a class of diterpen alkaloids e.g., Taxol (paclitaxel), 10-deacetyl baccatin III (DAB), baccatin III, cephalomannine, 10 deacetyl paclitaxel, 7-epi 10-deacetyl paclitaxel, 7-epi paclitaxel, etc.^1^. Among different taxanes, Taxol is known as the best drug for cancer treatment, particularly breast, ovarian, lung, head and neck cancer, and adenocarcinomas of the upper gastrointestinal tract^2^. Taxol in a very low quantity (0.004%- 0.1% of dry weight) have been mainly extracted from bark of yew (*Taxus* sp*.)* which is a very slow growing tree in turn^3^. On the other hand, total synthesis of Taxol is not economical either, because of its structural complexity^4^. Semi-synthetic approaches can provide Taxol, but still with plant-based precursors which are not enough nor their extraction is cost-effective. So, finding an achievable new source is indispensable^5^.

Recently endophyte fungi which colonize living plant tissues and most often have no pathogenic symptom there, have been introduced as alternative sources with enormous potential to produce bioactive compounds such as Taxol^6,7^. In comparison with the plants, they grow much faster and are more suitable for metabolic engineering. Easier extraction of Taxol from fungi would be worthwhile from both ecological and economic viewpoints^8,9^. There are reports showing that endophytes of Taxus *sp*.^10,11^, *Wrightia tinctoria*^12^, *Ocimum Basilicum,* and *Justicia gendarussa*^13^ are able to produce Taxol and their drivatives.

Extraction of taxanes from *Aspergillus niger* subsp. taxi isolated from yew and their application for chemical semi-synthesis of Taxol has been suggested ^1^. Ghaly et al.^14^ isolated *Alternaria alternata* from palm branches and detected 113.193 mg/L Taxol in its extract. So far more than 20 genera of endophytic fungi capable to produce Taxol have been detected, a few of which produced other taxanes as well^1,15^. *Corylus avellana* was the first plant from angiosperms that scientists discovered its ability to produce Taxol and their derivatives^3^. Taxan production by cultured hazel cells has been also well documented^16^. To the best of our knowledge however, comprehensive studies on the plausible ability of hazel endophytes has not been accomplished yet. The present resaerch was performed to evaluate the production capacity of taxanes by hazel endophytes. Here, we report a variety of important taxanes such as Taxol, DAB, baccatin III, and cephalomannine in noticeable amounts extracted from 18 genus of 5 classes of endophytic fungi isolated from *Corylus avellana*. Based on rapid growth, noticeable amounts of produced taxans, and low cost of cultivation and extraction procedure, these fungi could be considered as potential sources to provide precursors for semi-synthetic approach (Table 1).

**Table 1:** Location of sampling and the number of isolated endophytic fungi of *Corylus avellana* L. at each location.

Location						
Altitude (A.M.S.L.)	288	523	1430	1652	1680	2012
Coordinates	36° 59′ N, 49° 33′ E	37° 00′ N, 48° 44′ E	38° 23′ N, 48° 32′ E	36° 37′ N, 50° 11′ E	36° 48′ N, 50° 14′ E	34° 15′ N, 50° 59′ E
Soil pH	7.63	7.53	7	7.55	6.83	7.43
Number of isolates	20	13	52	44	78	42

## Results

### Isolation and growth of endophytes

**Figure 1:**
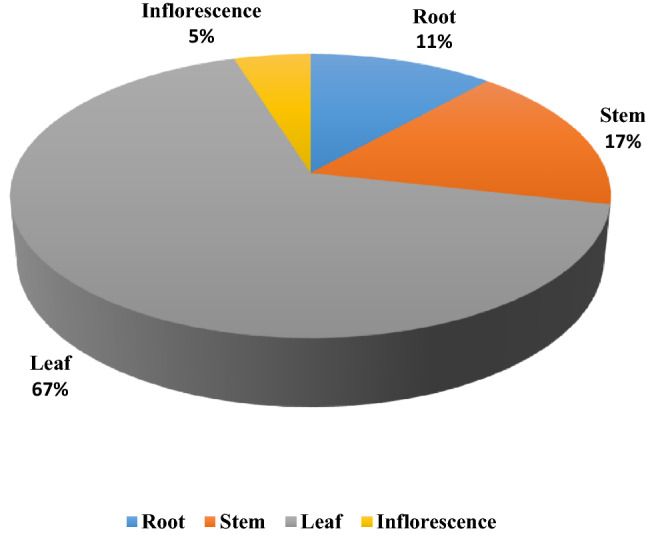
The percentage of endophytic fungi isolated from different organs of *Corylus avellana* L.

**Figure 2:**
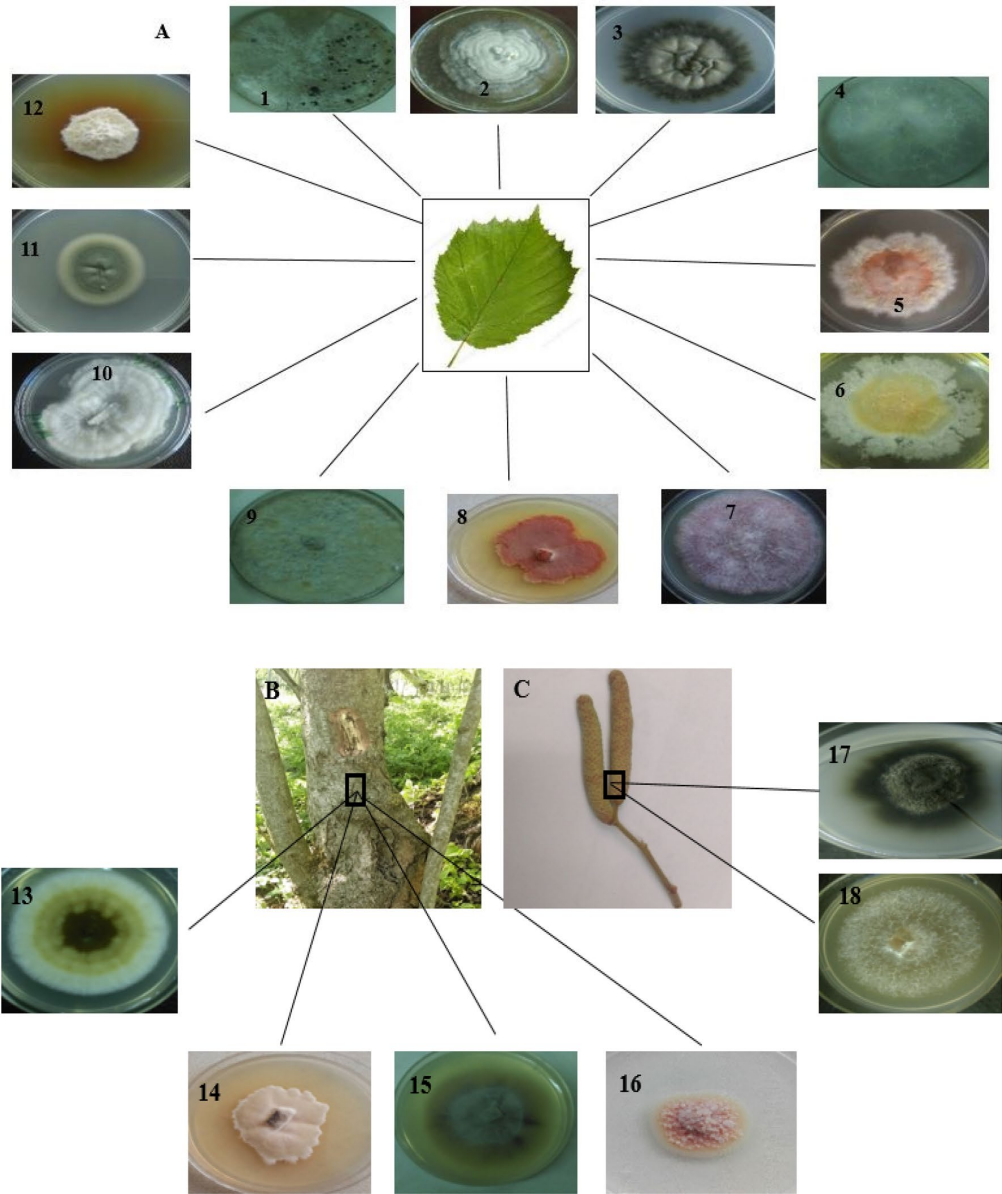
Colonies of endophytic fungi isolated from different parts of *C. avellana* L., (**A**) Leaf and 1–12 isolated fungi from it i.e., CA2*,* CA4*,* CA5*,* CA16*,* CA7*,* CA8, CA9, CA10, CA12, CA14, CA15, and CA18*,* respectively*.* (**B**) Bark and its isolated colonies (13–16) i.e., CA1, CA3, CA6, and CA13, respectively. (**C**) Inflorescence and colonies of two endophytes CA17 and CA11 isolated from it.

Two hundred forty-nine endophytic fungi were isolated from 1000 segments of different parts of *Corylus avellana* plant, most of them were isolated from leaves (Fig. 1). They were identified at first according to their culture characteristics on PDA (Fig. 2), and then were confirme based on ITS rDNA sequence analyses.

### Fungal genotyping

A total of 18 distinctive genotypes were detected at ≥ 98% sequence similarity threshold (Fig. S1). Except for CA13, which was basidiomycete, others belonged to the ascomycota (Table 2). These fungi belonged to four classes of the ascomycota phylum, including Dothideomycetes (eight species), Eurotiomycetes (three species), Leotiomycetes (one species), and Sordariomycetes (five species).

**Table 2:** Genotyping of Taxan producting endophytic fungi isolated from different section of *Corylus avellana* L*.*

Endophytic fungi	Accession number	Closest relatives in NCBI	ITS identity (%)	Phylum; class; order
CA**1**	MW296861	*Alternaria alternata* KT223359.1	99.24	Ascomycota, Dothideomycetes, Pleosporales
CA**2**	MW296850	*Arthrinium arundinis* MF476025.1	99.45	Ascomycota, Sordariomycetes, Xylariales
CA**3**	MW296859	*Aspergillus microcysticus* MH857716.1	100	Ascomycota, Eurotiomycetes, Eurotiales
CA**4**	MW296863	*Auxarthron alboluteum* KC253973.1	99.46	Ascomycota, Eurotiomycetes, Onygenales
CA5	MW296857	*Cladosporium variabile* MH863132.1	100	Ascomycota, Dothideomycetes, Capnodiales
CA6	MW296848	*Coniothyrium telephii* LT796830.1	98.52	Ascomycota, Dothideomycetes, Pleosporales
CA**7**	MW296853	*Cryptosporiopsis tarraconensis* EU707431.1	99.18	Ascomycota, Leotiomycetes, Helotiales
CA**8**	MW296852	*Epicoccum nigrum* MK388043.1	99.61	Ascomycota, Dothideomycetes, Pleosporales
CA**9**	MW296851	*Fusarium fujikuroi* MH857023.1	99.8	Ascomycota, Sordariomycetes, Hypocreales
CA1**0**	MW296854	*Fusarium tricinctum MN077464.1*	99.25	Ascomycota, Sordariomycetes, Hypocreales
CA**11**	MW296856	*Kalmusia variispora MG208006.1*	98.91	Ascomycota, Dothideomycetes, Pleosporales
CA1**2**	MW296849	*Melanconium hedericola* NR137940.1	98	Ascomycota, Sordariomycetes, Diaporthales
CA**13**	MW296855	*Meira geulakonigii* NR073344.1	99.31	Basidiomycota, Exobasidiomycetes, Exobasidiales
CA**14**	MW296847	*Neopestalotiopsis vitis* MH243066.1	99.62	Ascomycota, Sordariomycetes, Xylariales
CA15	MW296864	*Penicillium ruens* MN413162	97.82	Ascomycota, Eurotiomycetes, Eurotiales
CA**16**	MW296860	*Phoma crystallifera* KY977445.1	98.26	Ascomycota, Dothideomycetes, Pleosporales
CA**1**7	MW296858	*Ramularia mali KJ504778.1*	98.03	Ascomycota, Dothideomycetes, Capnodiales
CA1**8**	MW296862	*Stemphylium vesicarium* MH879836.1	98.59	Ascomycota, Dothideomycetes, Pleosporales

### Screening of taxan producing fungi

**Figure 3:**
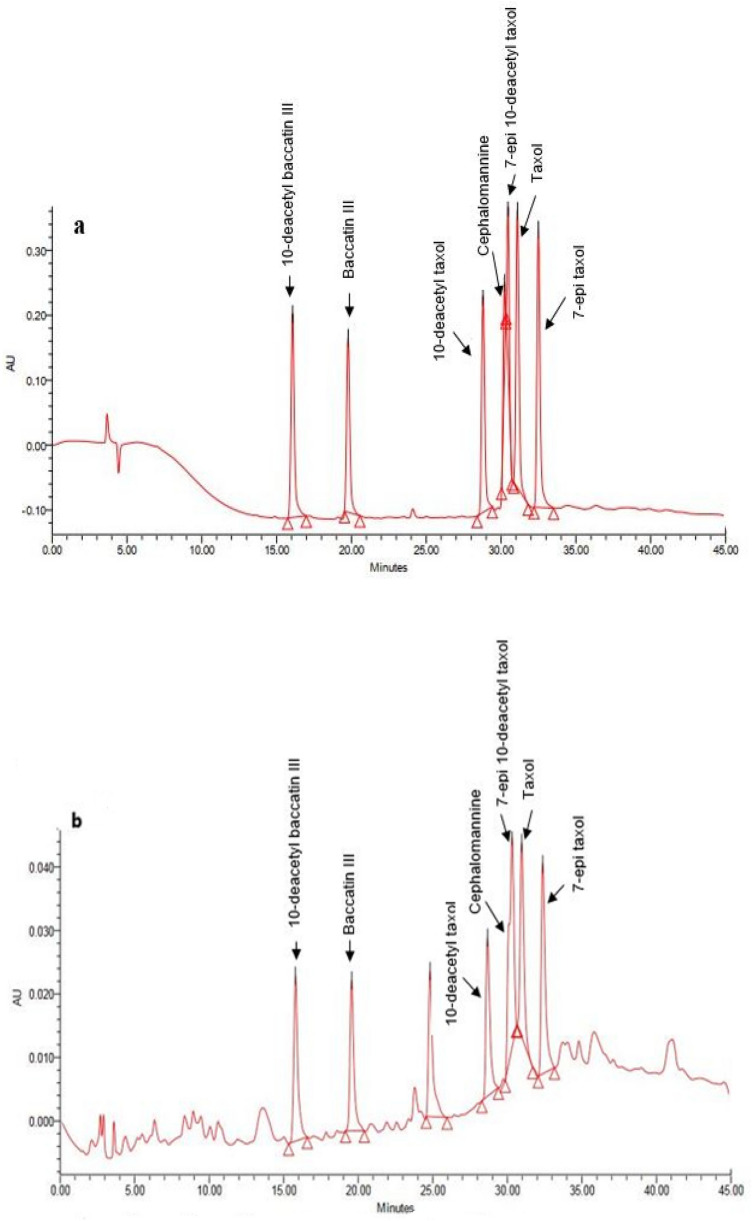
HPLC profile of taxanes standard (**a**) and CA7 as a representative of analyzed fungi (**b**).

Presence of toxoids in the extract of isolated endophytes was monitored by HPLC analysis (Fig. 3). Detected toxoids were structurally confirmed by LC–Ms (Fig. S2). The ]M + H[ peak calculated at, 545 m/z for DAB, 587 m/z for baccatin III, 812 m/z for 10 Deacetyl taxol and 7-epi 10-deacetyl taxol, 832 m/z for Cephalomanine, 854 m/z for Taxol and 7-epiTaxol (Fig. S2).

Eighteen isolated endophytes contained taxanes, among which five species i.e., CA12, CA3*,* CA16*,* CA18, CA2, showed remarkable total amounts of taxanes in solid media (Table 3). In addition, CA7 *,* CA15*,* CA2*,* CA18*,* CA3*,* CA11*,* and CA1 were the most taxan producing endophytes in liquid culture conditions (Table 4).

**Table 3:** The concentration of taxanes were quantified in endophytic fungi isolated from solid media. 10d BIII (10-deacetyl baccatin III); BIII (baccatin III); 10dT (10-deacetyl taxol); Ceph (cephalomannine); EDT (7-epi 10-deacetyl taxol), pacli (paclitaxel), 7 epi T (7-epi taxol); ND (not detected).

Endophytic fungi	10d BIII (mg/L)	BIII (mg/L)	10dT (mg/L)	Ceph (mg/L)	EDT (mg/L)	Pacli (mg/L)	7 epi T (mg/L)	Total (mg/L)
CA1	ND	0.1	0	0.1	0.1	0.1	1	1.4
CA2	16.4	ND	ND	ND	0.1	0.3	ND	16.7
CA3	20.4	ND	ND	ND	ND	ND	ND	20.4
CA4	6	ND	1.7	ND	ND	ND	0.4	8.1
CA5	ND	ND	ND	ND	0	ND	0.1	0.1
CA6	ND	0	ND	0	0	0	0.1	0.1
CA7	0.2	0.1	0.1	0	0	0.1	0.1	0.6
CA8	ND	ND	ND	0	0.1	0	0	0.1
CA9	ND	ND	0.2	0.1	0.1	0	0	0.4
CA10	ND	0	ND	0.1	ND	0	0	0.1
CA11	ND	ND	ND	0	0.1	ND	0.1	0.2
CA12	22.1	ND	ND	ND	ND	1	ND	23.1
CA13	ND	0	ND	0	0.2	ND	0	0.2
CA14	0.5	ND	ND	ND	0.1	ND	0	0.6
CA15	ND	ND	0.5	ND	ND	ND	1.1	1.6
CA16	ND	ND	11.8	ND	ND	ND	ND	11.8
CA17	ND	ND	ND	ND	0.1	0	ND	0.1
CA18	8.9	0.5	ND	ND	1.8	1.4	ND	12.6

**Table 4:** The concentration of taxanes in endophytic fungi isolated from *Corylus avellana*. 10d BIII (10-deacetyl baccatin III); BIII (baccatin III); 10dT (10-deacetyl taxol); Ceph (cephalomannine); EDT (7-epi 10-deacetyl taxol), pacli (paclitaxel), 7 epi T (7-epi taxol); ND (not detected).

Endophytic fungi	10d BIII (mg/L)	BIII (mg/L)	10dT (mg/L)	Ceph (mg/L)	EDT (mg/L)	Pacli (mg/L)	7 epi T (mg/L)	Total (mg/L)
CA1	2.4	0.4	ND	0.7	0.1	7.5	0	11.1
CA2	49	0.3	0.3	0.1	0.3	ND	0.2	50.2
CA3	9	0.2	ND	3	0	0.8	1.3	14.3
CA4	ND	0	0.1	0	0.5	0	0.2	0.8
CA5	ND	0	ND	1	0.2	0.1	0	1.3
CA6	ND	ND	ND	ND	0.1	ND	0.1	0.2
CA7	70.2	ND	0.1	0.8	0	0.1	2.8	74
CA8	ND	0.4	ND	ND	ND	0.6	ND	1
CA9	ND	1.1	ND	0.9	0	ND	0.5	2.5
CA10	4.2	0.1	ND	0	0.2	0	0.7	5.2
CA11	9.7	0	ND	0.2	ND	ND	0	9.9
CA12	ND	0.1	ND	ND	0.7	0	0	0.8
CA13	ND	0.1	ND	0.2	0	0	0	0.3
CA14	10.6	0.3	0.3	0.4	0.2	0.3	0.3	12.4
CA15	50.2	ND	0.2	0	0	0.1	0.1	50.6
CA16	ND	0.5	0.5	0.5	0.1	0	1.1	2.7
CA17	ND	0	ND	0.1	0.3	0.1	0.2	0.6
CA18	21.2	0.1	0.4	0.4	ND	0	0.9	23

The main taxoid of fungi that accumulated in solid and liquid media were DAB, followed by 10-deacetyl paclitaxel, cephalomannine, and Taxol, (Tables 3 and 4), and the highest alteration in the quantities of taxanes after transition from solid to liquid media was observed in DAB (Table 4). On the other hand, 7- epitaxol was detected in lower quantities than other taxanes in liquid media. Except for CA3*,* CA4*,* CA12 and CA16 whose total taxan contents were reduced in liquid media, for other species transfer to liquid culture was accompanied by significant increase of total taxan content and induction of certain taxoids (Table 4). As an instance, baccatin III that was not observed in solid culture of CA2, CA3*,* CA8*,* CA9*,* CA16*,* was remarkably induced by transferring the fungi into liquid medium. Likewise, cephalomannine was not detected in solid culture of CA3*,* CA7*,* and CA16 but dramatically induced in liquid media (Tables 3 and 4). It is noteworthy however that the potential to produce a given taxoid compound was not equal for all isolated endophytes. For example, CA7 produced the greatest amount of DAB (70.2 mg/L), while CA16 showed the highest 10-deacetyltaxol (11.8 mg/L) and CA1 produced the greatest amount of Taxol (7.5 mg/L) (Table 4).

During 21 days growth in liquid culture, the endophytes released 9- 90% of Taxoids into their media (Table 5). The most specific yield of toxoids respectively belonged to CA7, CA2,and CA15, while the specific yield of other endophytes were much lower (Table 5).

**Table 5:** Intracellular and extracellular contents and percentages of taxans release from isolated endophytic fungi.

Endophytic fungi	Intracellular taxan (mg/L)	Extracellular taxan (mg/L)	Release (%)	Specific yield of taxanes (µg/gDW)
CA1	10.3	0.8	7.2	18.5
CA2	17.5	32.7	65.1	167.3
CA3	2.1	12.2	85.3	22
CA4	0.3	0.5	62.5	2.7
CA5	1.1	0.2	15.3	1.9
CA6	0.1	0.1	50	0.2
CA7	51.3	22.7	30.6	211.1
CA8	0.6	0.4	40	4
CA10	4.9	0.3	5.7	3.6
CA9	1.8	0.7	28	5.2
CA11	0.1	9.8	98.9	11
CA12	0.3	0.6	75	1.1
CA13	0.2	0.1	33.3	1.2
CA14	0.8	11.6	93.5	23.1
CA15	26	24.6	48.6	56.2
CA16	2.1	0.6	22.2	4.5
CA17	0.3	0.4	57.1	1.2
CA18	0.9	22.1	96	46

**Figure 4:**
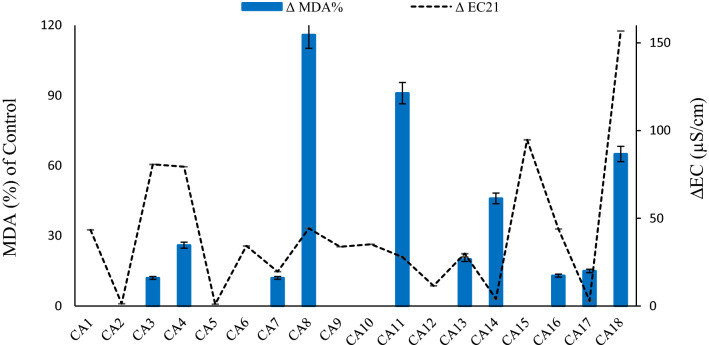
The changes of mycelial membrane lipid peroxidation and medium electro-conductivity of isolated fungi after 21 days growth in suspension culture, compared to day 0. No column is shown whenever there was no relative change of MDA or EC, otherwise the changes were statistically significant at a level of *P* ≤ 0.05, based on Student’s *t* test.

Changes of mycelial membrane lipid peroxidation rate and conductivity of the medium are shown in Fig. 4. Electrolyte leakages of CA3*,* CA4*,* CA8*,* CA11*,* and CA18 increased during growth in liquid media and it was parallel with increase of their MDA (Fig. 4). For other endophytes however, no conclusive relationship was observed between EC and MDA (Fig. 4).

### Cytotoxicity of the extracts of isolated endophytes

**Figure 5:**
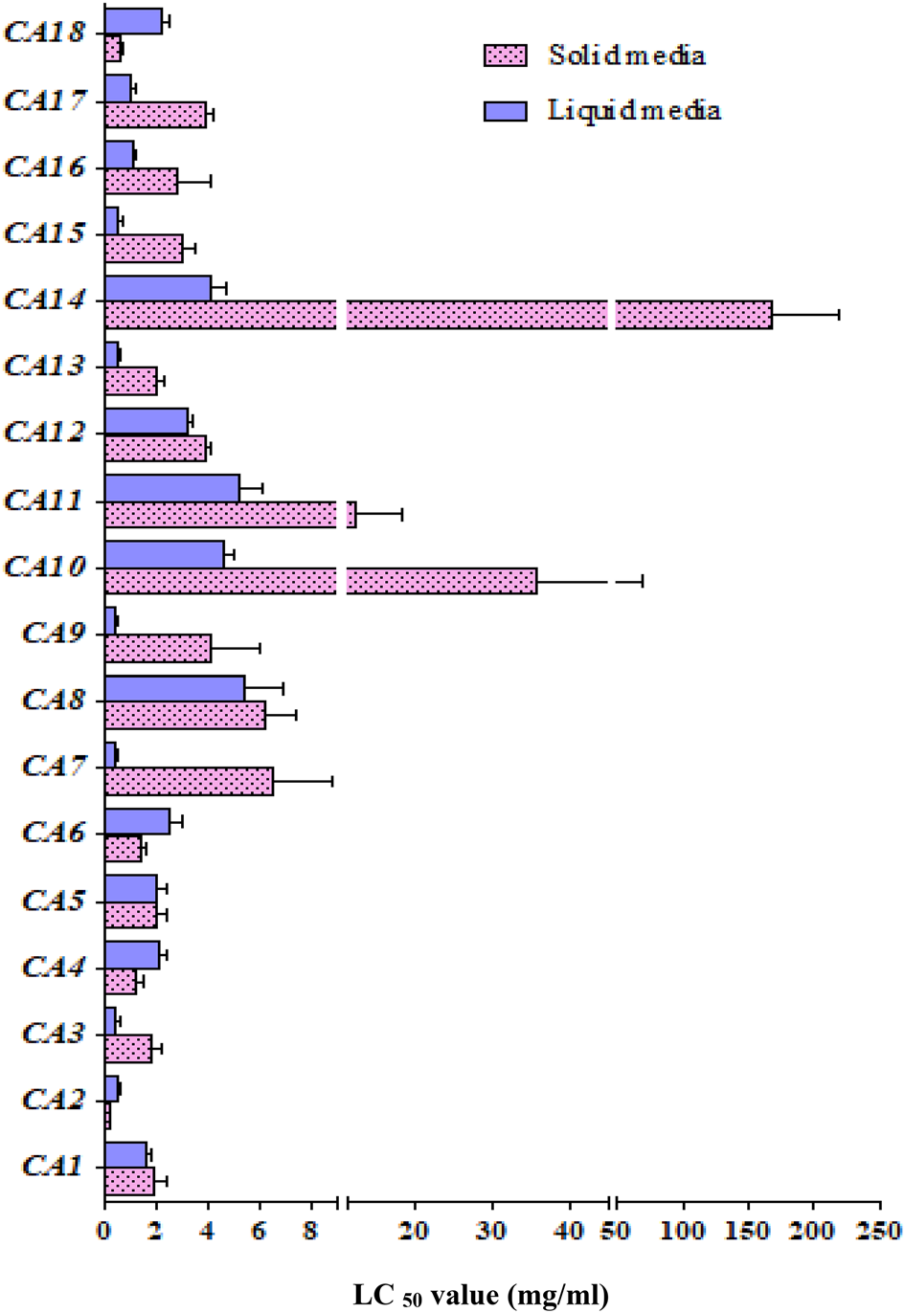
LC_50_ values of the extracts of taxan-producing fungi grown in solid and liquid media.

Evaluation of cytotoxicity of isolated endophytes on MCF-7 breast cancer cells showed that the extract of almost all fungi had potential to inhibit the growth of cancer cells (Fig. 5). Except for CA2*,* CA6*,* CA12*, and* CA18 cytotoxicity of suspension media grown fungi were higher (lower LC_50_) than those in solid ones (Fig. 5). In liquid media, CA1 and CA12 showed the most and the lowest inhibitory potential, respectively (Fig. 5). In solid cultures, CA2 and CA14 showed the highest and the lowest inhibitory effects, respectively (Fig. 5).

## Discussion

Considering faster growth rate and ease of extraction, Taxan-producing fungi could be renewable resource for commercial production of Taxol, compared to Taxol producing plants^1^. So far, more than 200 Taxol producing endophytic fungi from different gymnosperm and angiosperm hosts have been introduced^17^. Salehi et al.^18^ isolated one endophyte from *Corylus avellana*. However, Taxol production was not stable in that fungus after 6 sub-cultures. Here, the presence of 7 taxoids is reported in 18 isolated endophytes from *C. avellana*. It is noteworthy that production of not only Taxol but also other taxoids i.e., DAB, baccatin III, cephalomannin, 10 deacetyl taxol, 7-epi 10-deacetyl taxol, 7-epi taxol by these fungi was stable even after ca. 30 subculture, as confirmed by HPLC and LC-Mass analysis. Most of endophytes were isolated from leaves, probably because of less infection barriers exist in leaves^19^. It is well documented that antibiotics, anticancers and other bioactive molecules are predominately produced by fungi that belong to the Pezizomycotina ascomycete class, and several basidiomycete classes^20^. In the present study also more than 94% taxan producing endophytes were ascomycete and one species, CA13*,* was basidiomycets. The latter is introduced for the first time as a taxan bearing basidiomycete.

Most of previous researches focused on the detection of the most known taxoids i.e., Taxol, DAB, and baccatin III^1,21^. Taxol has already been reported in CA5, CA8*,* CA16*,* CA18, and also was detected in the present research^22–25^. CA7*,* CA12*,* CA2*,* CA17*,* CA4*,* CA6*,* and CA11*,* however, are ascomycetes reported here for the first time, not only for Taxol production but also for other taxanes.

Transferring fungi from solid to suspension media noticeably induced the production of certain taxanes. This can be attributed to homogenous dispersion, higher availability, and subsequent absorption of micro/macro nutrients, and stimulation of expression/activation of enzymes involved in taxan biosynthesis pathway in suspension cultures^26^.

The most alterations of taxanes in liquid media were observed in DAB content of CA15 and CA7*,* and Taxol of CA1*.* DAB is the precursor of baccatin III, but its increase in CA15 and CA7 in liquid medium did not lead to increase of baccatin III. Conversion of DAB to baccatin III is catalyzed by the activity of 10-deacetylbaccatin III-10β-O-acetyltransferase (DBAT). Studying Taxol biosynthesis in developing *Taxus baccata* plantlets, Onrubia et al.^27^ observed that despite the increase of DAB the expression of DBAT gene was low and baccatin III did not increase. It suggests that the increase of the substrate DAB does not necessarily lead to increase of the product, baccatin III.

Secondary metabolites produced by plant cells are stored in vacuoles, however, because of their limited capacity, these compounds bypass membrane barrier and release to the medium. One may conclude that the more damaging membrane, the more release of secondary compounds. In the present study a positive correlation was observed between increase of electrolyte leakages and membrane lipid peroxidation rate with percentage of taxan release in CA3*,* CA4*,* CA8*,* CA11*,* and CA18*.* Likewise, in CA1*,* CA5*,* CA10 lower release of taxanes was in parallel with lower rates of EC and MDA changes. However, it cannot be concluded for all, it has been suggested that taxanes may secrete via hydrophobic bodies exocytosis process from living hyphae, not dead cells^28^. So, the underlying mechanism of accumulation or release of taxans in remained endophytes needs to be clarified by further investigations.

The LC_50_ values of the extracts of most of endophytes in suspension media were lower than solid media. It may be expectable because more taxanes were induced in liquid medium than the solid one. Interestingly in CA2*,* CA6*,* and CA18 LC_50_ was lower in solid cultures, where the content of paclitaxel was higher than suspension cultures. This implies that compared to other taxanes, paclitaxel content of endophytes extract has a more determinant role in cytotoxicity against MCF7 cancer cells. Nevertheless, no clear relationship was observed between the amount of a specific taxoid compound and the LC_50_ of the extract. This may be explained, at least in part, by the presence of various cytotoxic compounds with various relative amounts in fungi extract or low quantity of taxans. Trying to elucidate molecular mechanism of cytotoxicity of Taxol in anaplastic thyroid cancer cells, Pushkarev and co-workers found that the drug at low concentrations can activate a variety of signaling cascades, both proapoptotic and prosurvival^29^. Moreover, in the present study the inhibitory effects of endophyte extracts were tested on MCF-7 cell line while effectiveness of chemotherapeutic drugs is not the same neither for all compounds nor all types of cancer^30^.

The results presented here clearly showed that endophyte fungi isolated from *Corylus avellana* leaves are potent candidates for extraction of Taxol and its precursors particularly 10-DAB and baccatin III. The former is currently isolated from yew spp. as precursor for large-scale production of paclitaxel via semi-synthesis^1^. Due to the frequency of hazel plantations in the world, wide verities of its endophytes and the most natural content of DAB, it is likely that these fungi can overcome the shortage of natural Taxol and its precursors. Metabolic pathway of taxanes, corresponding genes, and their similarity or divergence with those of *Taxus* sp. and *Corylus avellana* are needed to be clarified by further investigations.

## Methods

### Plant samples collection, isolation and growth conditions of endophytes

All methods were carried out in accordance with the relevant institutional, national, and international guidelines and legislation. Besides they were discussed and approved by the Research Ethics Committee of Tarbiat Modares University. The plant samples were collected from six local gardens in Iran with different altitudes (Table 1). Permissions or licenses to collect different parts of the plants were obtained from owners. The plants were identified and authenticated at first by authors based on Colorful Flora of Iran^31^ and confirmed by Dr Vali-allah Mozaffarian of the Institute of Forests and Rangelands Research,Tehran, Iran and a voucher specimen deposited at the herbarium of the institute.

The samples were picked from mature trees, put in labeled sealed plastic bags, and stored at 4 °C until isolation of their endophytes.

After washing with detergent and rinsing, the samples were surface sterilized via sequential washing with EtOH 70% (1 min) and NaOCl (containing 5% active chlorine) (15 min) followed by three times rinsing with sterilized distilled. Discrimination between endophytes and air born contamination was achieved by preparing some plates without sample and checking the colonies, if any. The sterilized samples were cut into small pieces (0.5 × 0.5 cm) and removed on potato dextrose agar (PDA) media. The plates were incubated at darkness at 27 ± 2 °C. Emergence of hyphae were checked daily. In order to check their purity, mycelia tips were picked and removed on 2% agar containing distilled water without PDA. Hyphal tips were picked from pure colonies and re-transferred on PDA media for further identification and chemical assays^32^. The media were renewed every three weeks. Frequent subcultures on PDA assured us that taxans originated from host plants were depleted.

For suspension culture, the pure fungi were transferred into 500 mL Erlenmeyer flasks containing 150 mL of potato dextrose broth (PDB) and agitated on reciprocal shakers (120 rpm) in darkness, 27 ± 2 °C for 21 days. The cultures were filtered under reduced pressure and both filtrates and mycelia were extracted for taxoids. Growth status of endophytes in liquid media was monitored by measuring electro-conductivity of the filtrate and the mycelial membrane integrity was manifested by malondealdehyde (MDA) as the product of rate of membrane lipid peroxidation^33^.

### Detection of Taxan-producing fungi and molecular techniques

Taxanes were extracted from both filtrate and fugal residue. The latter was homogenized in 10 mL MeOH followed by centrifugation at 5000×*g* for 10 min. A mixture of CH_2_Cl_2_ and water (1:1) was added to the supernatant, mixed, and centrifuged again. Methylene chloride phase was collected, air-dried, re-dissolved in 200 µL of MeOH and filtered with a 0.2 µm syringe filter before applying for HPLC analysis.

Equal volume of MeOH was added to the filtrate, kept overnight and then evaporated. Next, a mixture of CH_2_Cl_2_ and water (1:1) was added and the procedure was followed as above. For qualitative and quantitative assay, a HPLC system (Waters, e2695, USA) equipped with C18 column (Perfectsil Target ODS3, 5 μm, 250 × 4.6 mm, MZ-Analysentechnik, Mainz, Germany) was used. Elution was conducted at a flow rate of 0.8 mL min^−1^; mobile phase was water (containing 0.1% CH_3_CN): MeOH. Taxanes were eluted by a gradient mode composed of 0–30 min a linear gradient of 40–78% MeOH followed by an isocratic elution with 78% MeOH for 30–40 min, and finally (40–45 min) the decrease of MeOH to 40%^34^. The retention time and peak area of genuine taxan standards were used for identification and quantification of taxanes (Sigma-Aldrich, USA; ChromaDex, USA). For more reassurance, each peak was spiked by injection of its corresponding standard.

The samples were also analyzed by an Agilent 6410 HPLC system coupled to a triple quadrupole ion trap mass spectrometer, equipped with an electrospray ion source. The column was an Eclipse C18 (3.5 lm particle size, 100 mm length, and 4.6 mm width). The flow rates of solvent and injection volume were 350 µL/min and 50 µl/min, respectively. Mobile phase A was distilled water: MeOH (70:30, v/v), and mobile phase B consisted of 0.1% formic acid-containing MeOH. An isocratic flow (1 mL/min) was used for 5 min. The gradient, then, started at 100% A and linearly increased to 100% B over the course of 25 min, followed by an isocratic gradient of 100% B for 10 min, (total run time 40 min). Mass spectra were acquired in product ion mode. ESI/MS verified the presence of taxanes in samples in a product ion mode according to structurally diagnostic ions in the LC–MS spectra^35^.

### Identification of isolated fungi

The preliminary identification was done based on the (macro- and micro morphology of the fungal colony and the characteristics of the hyphae, spores and conidia produced by isolate fungi^36–43^. For molecular analysis, total DNA was extracted using cetyl trimethylammonium bromide (CTAB). Aliquots (1g) of purified fungi were ground, transferred to a sterilized Eppendorf tube and 700 µL buffer containing 2% (W/W) CTAB was added. The mixture was incubated for 30 min at 55 °C and then 700 µL chloroform and isoamyl alcohol (24:1, v/v) were added. The total DNA was precipitated by addition of 700 µL cold isopropanol. Finally, The DNA pellet was washed with 70% (v/v) EtOH, precipitated by centrifugation, dried at 37 °C, and re-dissolved in 100 µL TE (10 mM tris containing 1 mM EDTA) buffer. For genotyping, partial gene sequence of the nuclear ribosomal internal transcribed spacer region were used^44^. ITS rDNA, including ITS1, 5.8S, and ITS2 regions were amplified using the primers ITS1 (5ʹ-TCCGTAGGTGAACCTGCGG-3ʹ), ITS1f (5'-CTTGGTCATTTAGAGGAAGTAA-3ʹ) and ITS4 (5ʹ-TCCTCCGCTT ATTGATATGC-3ʹ).

Finally, the PCR products of ITS genes were run on 1% agarose gel. Subsequently, PCR amplicons were sequenced by Genetic Codon Company (Tehran, Iran). To identify fungi, sequences were compared with those available corresponding sequences in Gene Bank database using the BLASTn program (http://www.ncbi.nlm.nih.gov). The sequences obtained were submitted to the GenBank database with accession numbers from MW296847 to MW296864. GTR + I + G was selected as the best-fit model and the Bayesian analyses were performed using MrBayes v. 3.2.6 for phylogenetic tree retrieval^45^.

### Evaluation of cytotoxicity of fungal extracts to MCF-7 cell line

Inhibitory effects of fungal extracts on cancerous cells were tested using MTT salt (3-(4, 5-dimethylthiazol-2-yl)-2,5-diphenyltetrazolium bromide) assay. The percentage of cell viability was obtained by deducting the absorbance of control from the sample, dividing the absorbance of control. Lethal concentrations (LC50) were calculated by Graph Pad Prism (5.04) software^35^.

### Statistical analysis

All observations and experiments were repeated at least 3 times with 3 independent replicates. Statistical analysis was performed using Student’s *t* test, and the differences between the treatments were deemed significant at a level of *P* ≤ 0.05.

### Consent for publication

All authors read the MS and are agree with submission in its present form to the journal of Scientific Reports. The MS is original and is not considered for publication by other journals.

## Supplementary Information

Supplementary Information.
