# Supplementary material for: Taxoid profile in endophytic fungi isolated from Corylus avellana, introduces potential source for the production of Taxol in semi-synthetic approaches
Source: Sci Rep. 2022 Jun 7;12:9390. doi: 10.1038/s41598-022-13602-6 (PMC9174271; doi:10.1038/s41598-022-13602-6)
Supplement: Supplementary file 1 — Supplementary Information. [file 41598_2022_13602_MOESM1_ESM.docx]

**Fig. S1.** Molecular identification of taxan-producing fungi based on ITS rDNA sequence information with Bayesian analyses.


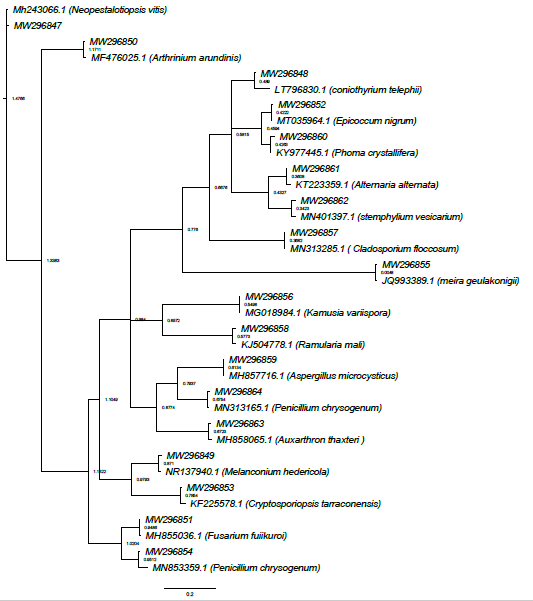


**Fig. S2**. LC-MS analysis of standard Taxan (a,b,c,d,e) and fungal taxan (f,g,h,i,j). The ]M+H[ peak calculated at, 545 m/z for DAB, 587 m/z for Baccatin III, 812 m/z for 10 Deacetyltaxol and 7-Epi 10-deacetyl taxol, 832 m/z for Cephalomanine, 854 m/z for Taxol and 7-epiTaxol. The samples were dissolved in methanol and injected using a spray flow of 1µL/min.


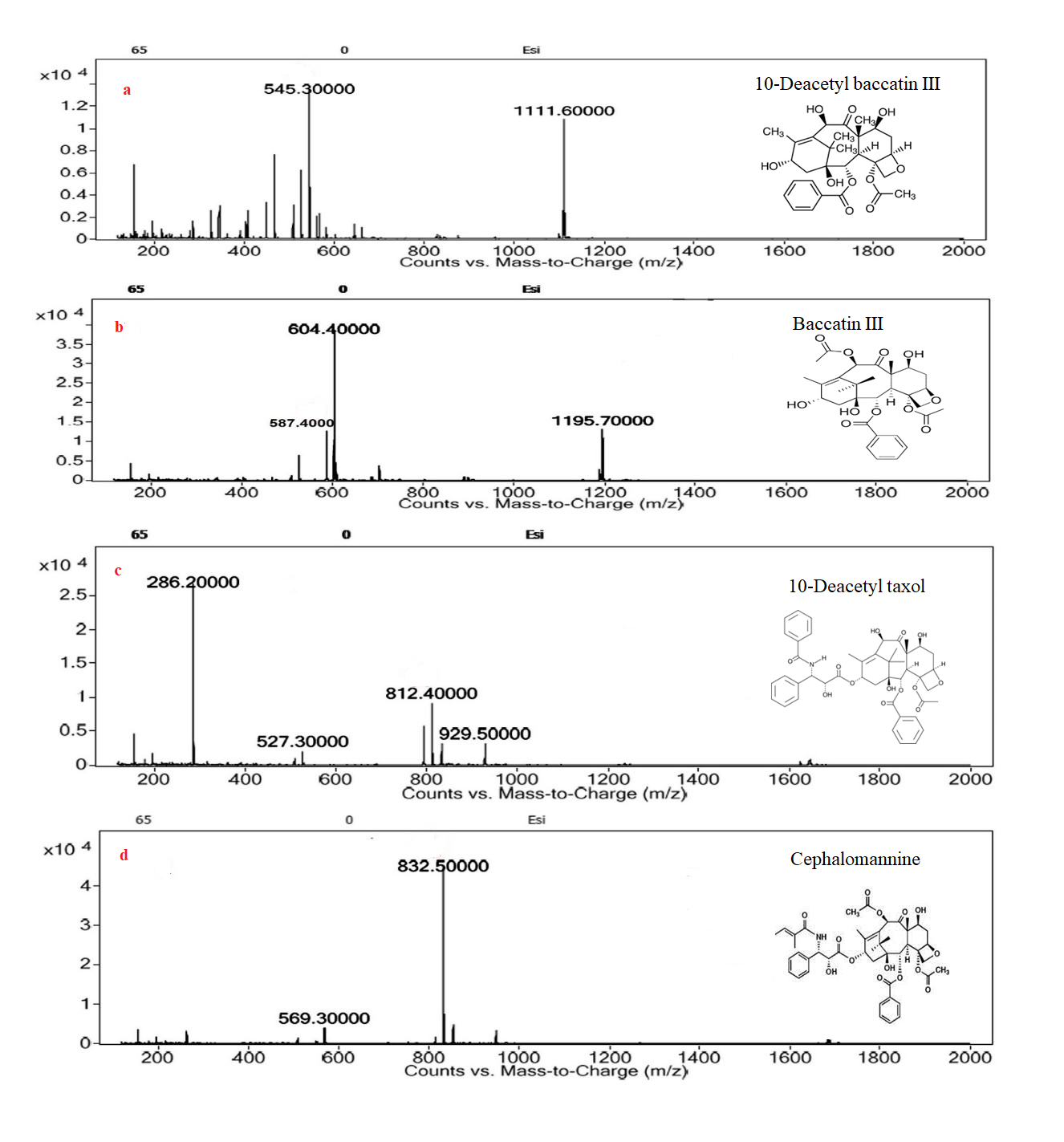


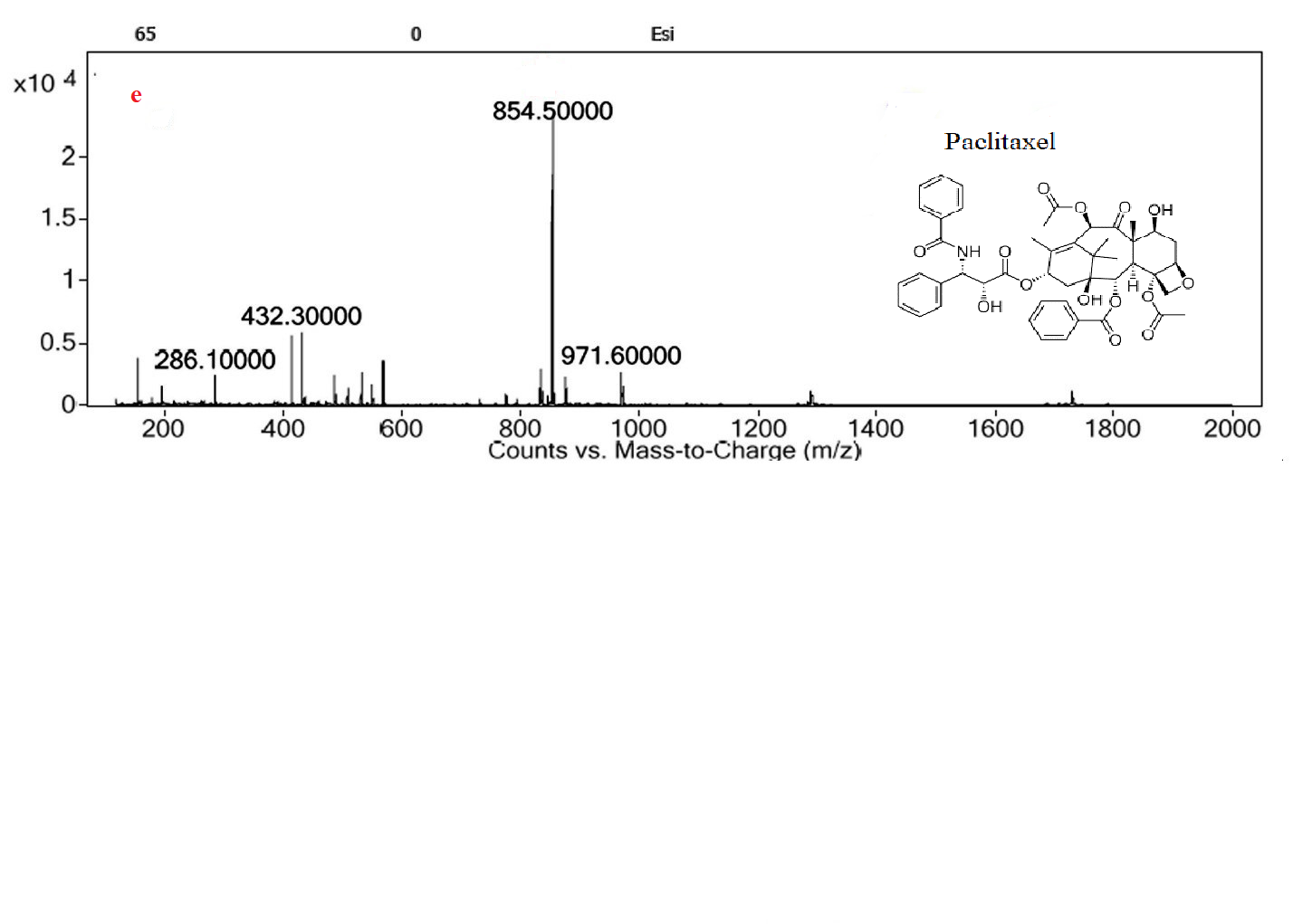

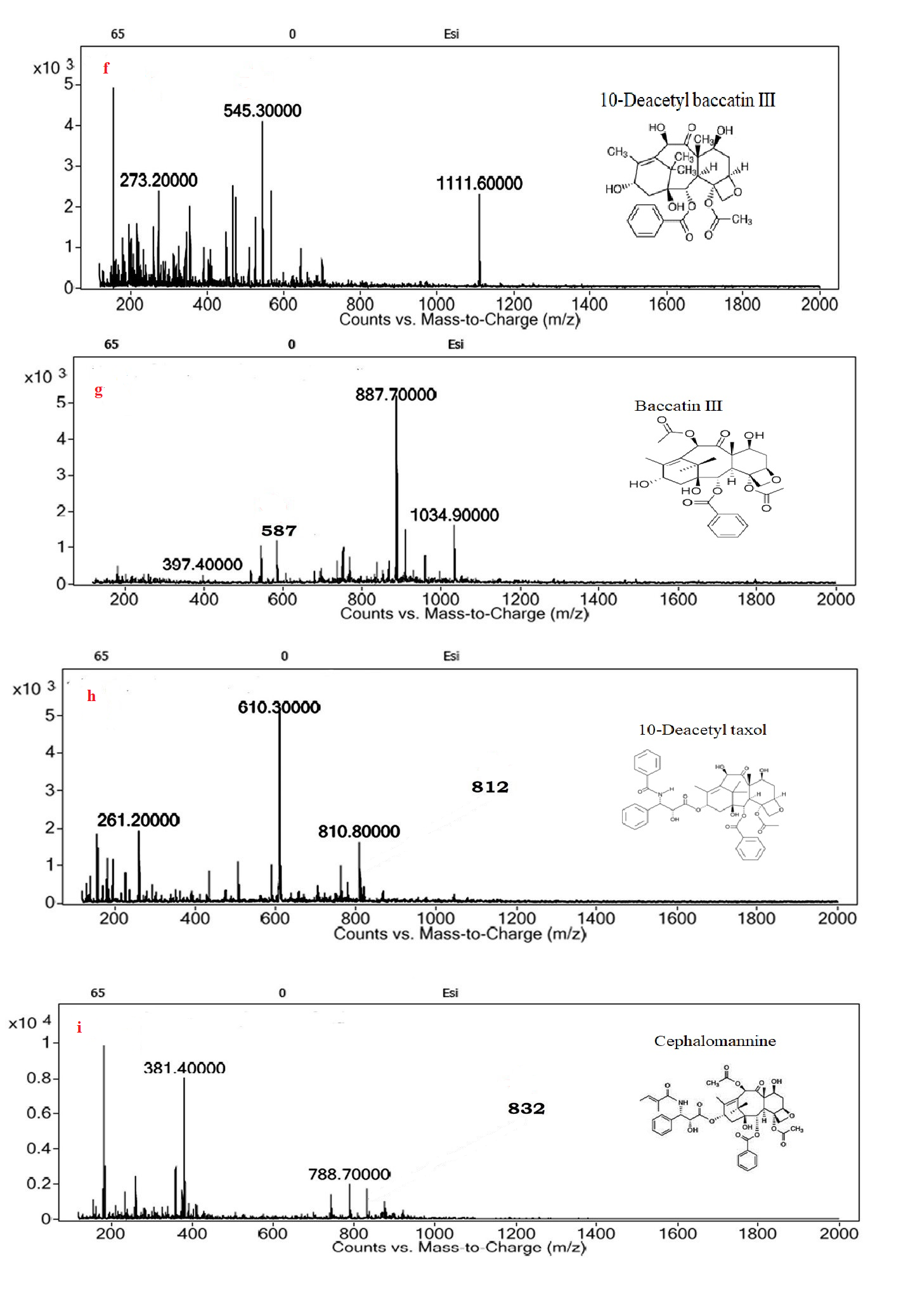


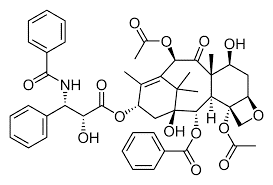

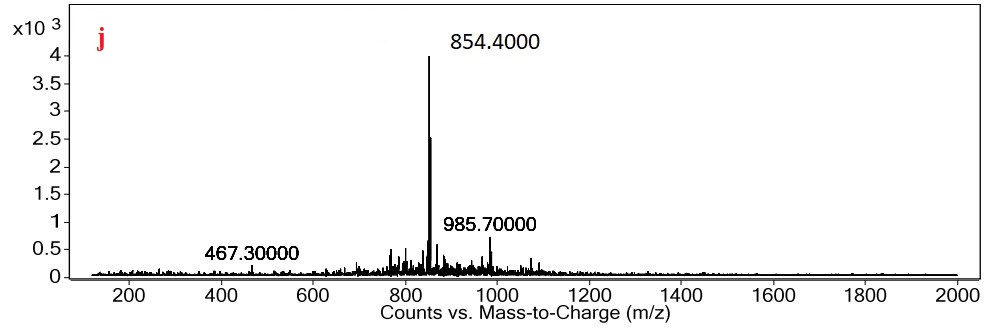


Paclitaxel
